# Walking bumblebees see faster

**DOI:** 10.1098/rspb.2023.0460

**Published:** 2023-05-31

**Authors:** Lisa Rother, Robin Müller, Erwin Kirschenmann, James J. Foster, Sinan Kaya-Zeeb, Markus Thamm, Keram Pfeiffer

**Affiliations:** ^1^ Department of Behavioral Physiology and Sociobiology, Biocenter, University of Würzburg, 97074 Würzburg, Germany; ^2^ Department of Biology, University of Konstanz, 78457 Konstanz, Germany

**Keywords:** electroretinograms, photoreceptors, *Bombus terrestris*, state dependency, temperature, Gaussian white noise

## Abstract

The behavioural state of animals has profound effects on neuronal information processing. Locomotion changes the response properties of visual interneurons in the insect brain, but it is still unknown if it also alters the response properties of photoreceptors. Photoreceptor responses become faster at higher temperatures. It has therefore been suggested that thermoregulation in insects could improve temporal resolution in vision, but direct evidence for this idea has so far been missing. Here, we compared electroretinograms from the compound eyes of tethered bumblebees that were either sitting or walking on an air-supported ball. We found that the visual processing speed strongly increased when the bumblebees were walking. By monitoring the eye temperature during recording, we saw that the increase in response speed was in synchrony with a rise in eye temperature. By artificially heating the head, we show that the walking-induced temperature increase of the visual system is sufficient to explain the rise in processing speed. We also show that walking accelerates the visual system to the equivalent of a 14-fold increase in light intensity. We conclude that the walking-induced rise in temperature accelerates the processing of visual information—an ideal strategy to process the increased information flow during locomotion.

## 1. Background

It is well documented that the processing of visual information is adjusted to the behavioural state in humans [1], rodents [2–7] and insects [2,8]. Motion-sensitive neurons in flies increase their gain during walking [9], flying [10] and movement of the halteres [11], and these locomotion-dependent changes are mediated by octopamine [12,13]. However, it remains unclear if the behavioural state affects only the neuronal processing, or if locomotion might also alter sensory processing at the photoreceptor level. Previous studies have shown that photoreceptors of different insect species respond faster at higher temperatures [14–18]. Bumblebees, like other insect species, are capable of regulating their body temperature over a wide range of temperatures independent of the ambient temperature by producing heat using their flight muscles. The heat generated in the thorax is then passed to the head by anterograde pumping of haemolymph [19]. It has therefore been suggested that insects that are capable of thermoregulation might be able to use this ability to increase the temporal resolution of their visual systems [18]. This would be similar to some species of large predatory pelagic fish that are able to keep their eye temperature 10–15°C above the ambient water temperature, which results in increased temporal resolution of their visual system [20,21]. However, direct experimental evidence showing that locomotion increases the processing speed of the visual system in insects has been lacking so far. By comparing electroretinograms (ERGs) from tethered bumblebees that were either walking or sitting, we provide here the first evidence that locomotion indeed speeds up the visual processing in bumblebees.

## 2. Methods

### (a) Animals

All experiments were performed using worker bumblebees (*Bombus terrestris*). Colonies were obtained from a commercial supplier (Biobest Group NV, Westerlo, Belgium) and kept either in climate chambers at 25°C and 55% RH under 12L : 12D or in flight arenas in a laboratory room with windows. Animals were provided with ad libitum food (pollen (Naturwaren-Niederrhein GmbH, Goch, Germany) and API-invert (Biobest Group NV, Westerlo, Belgium)).

Prior to electrophysiological recordings, bumblebees were placed in the freezer (−15°C) for 5–20 min to immobilize them. The animals were then waxed to a three-dimensional-printed holder using dental wax (Omnident, Rodgau, Germany). Head and anterior edge of the thorax were waxed to the holder to prevent head movement-induced recording artefacts, while legs and abdomen were free to allow walking.

ERGs were recorded differentially, using two silver wires (Ø 0.075 mm, Advent Research Materials Ltd, Eynsham, UK) as recording electrodes. Prior to insertion of the electrodes, the cornea of each compound eye was punctured with the tip of a minute pin (Ø 0.2 mm, Ento Sphinx s.r.o., Pardubice, Czech Republic). To reduce contamination of the ERG signal with lamina potentials, care was taken to insert the electrodes just deep enough to establish electrical contact with the photoreceptor layer. In all recordings, the recording sites were in the middle of the dorsolateral extent of the eyes at the laterally facing ommatidia. The insertion site was sealed with petroleum jelly to protect the eye from desiccation and to stabilize the electrodes mechanically. At the end of the preparation, the reference electrode (silver wire, Ø 0.25 mm, World Precision Instruments, Sarasota, USA) was inserted into the head capsule at the posterior dorsal edge of the head just outside the compound eye.

### (b) Electroretinograms

All experiments were carried out at ambient temperatures of 25°C. The extracellular signals were high-pass filtered at 1 Hz and amplified 100 x with an ELC-01MX amplifier equipped with a differential miniature headstage (npi electronic, Tamm, Germany), digitized with a Power 1401 (Cambridge Electronic Design, Cambridge, UK) at 3 kHz and recorded with Spike2 version 9 (Cambridge Electronic Design, Cambridge, UK).

### (c) Behavioural set-up

**Figure 1.**
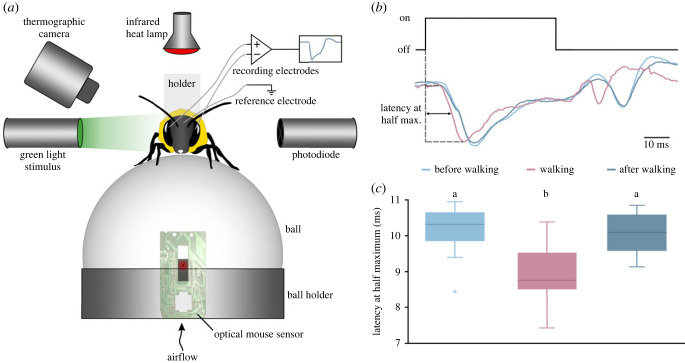
Experimental set-up and ERGs of sitting and walking bumblebees stimulated with green light pulses. (*a*) Schematic illustration of the experimental set-up. The bumblebee was tethered and positioned on top of an air-supported ball. While presenting green light (530 nm) pulses, the ERG was recorded. An infrared lamp and a thermographic camera were used to control and measure the temperature. (*b*) Upper trace: single pulse of green light (50 ms duration at 1 Hz, pulse intensity: 2 × 10^15^ photons cm^−2^ s^−1^). Lower panel: ERG response curves of one example trace of an animal during walking (violet) or sitting (light blue: before walking, dark blue: after walking). The time difference between the onset of the light pulse and the crossing of the half-maximum amplitude gave the latency. (*c*) Boxplots of the latency at half-maximum of the ERG response during walking (violet) or sitting (light blue: before walking, dark blue: after walking) of 16 bumblebees. The latency at half-maximum was significantly shorter during walking than during sitting (*p* < 0.001, Wilcoxon test with Bonferroni correction), but no differences were seen when comparing before and after walking (*p* = 0.1359, Wilcoxon test with Bonferroni correction). Boxplots show median, interquartile range (IQR), whiskers with 1.5 x IQR and outliers greater than 1.5 x IQR.

Tethered bumblebees were held by a micro-manipulator and positioned to sit in a natural posture on an air-supported Styrofoam ball (diameter: 50 mm) in the set-up. The forward motion and rotation in the yaw axis of the ball were registered using an optical mouse sensor connected to an Arduino Due and transferred to the CED 1401 as an analogue signal. The temperature of the animals' eye was continuously monitored using a thermographic camera (FLIR A65, lens: 45°, *f* = 13 mm, FLIR, Wilsonville, USA). The thermographic data were recorded, stored and extracted using FLIR software. A schematic view of the complete experimental set-up is shown in figure 1*a*.

### (d) Stimulation

For visual stimulation, light from a green LED (Osram Oslon SSL 80 LT CP7P, dominant wavelength: 528 nm, full width at half-maximum: 33 nm) was focused onto one end of a light guide using a fibre coupling lens (Carclo 10356, Carclo, Slough, UK). The other end of the light guide was positioned 9 cm away from the eye of the animal. The diameter of the light guide was 4 mm, resulting in a circular stimulus subtending a visual angle of 2.5°. In each preparation, the light guide was directed at the recording site such that the ERG amplitude in response to a light pulse was maximal. The current through the LED was controlled by a custom-built voltage-to-current converter, which was driven by an analogue output of the CED 1401. Stimulus intensity was calibrated using a spectrophotometer (Maya 2000 Pro, Ocean Optics). During experiments, light stimuli were monitored by recording the output from an integrated photodiode/transimpedance amplifier, which was placed across from the light guide. In the first set of experiments, animals were stimulated with light pulses (50 ms duration at 1 Hz, pulse intensity: 2 × 10^15^ photons cm^−2^ s^−1^). In the second set of experiments, we used Gaussian white noise as a stimulus. A 10 s Gaussian white noise signal was created using the open-source software Audacity (www.audacityteam.org) and saved as a .wav file, which was then imported into Spike2. The noise was then low-pass filtered using the Spike2 implementation of a nine-pole Butterworth filter with a corner frequency of 250 Hz. This gave a noise stimulus with a flat power spectrum up to approximately 220 Hz (see electronic supplementary material, figure S1). Absolute intensity of the stimulus was adjusted using neutral density filters. In most recordings, the mean intensity of the noise stimulus was set to 1.74 × 10^14^ photons cm^−2^ s^−1^. To test the intensity dependence of the ERG response three additional mean intensities were used: 7.27 × 10^14^ photons cm^−2^ s^−1^, 1.31 × 10^15^ photons cm^−2^ s^−1^ and 2.41 × 10^15^ photons cm^−2^ s^−1^. Prior to testing, animals were adapted for at least 10 min with the intensity that was to be used during testing. To compare different states of locomotion, the animals first sat on the Styrofoam ball without walking, then were encouraged to walk by gentle brush strokes on the abdomen (some animals started walking without encouragement) and at the end sat again. Visual stimulation was continuous throughout each experiment.

To test the effect of externally applied heat, an infrared heating lamp was turned on until the animals’ eye had a temperature of about 37°C. Then, the heat lamp was turned off and recording was continued until the initial head temperature was reached.

### (e) Quantification and statistical analysis

Calculation of cross-correlation and evaluation of responses to light pulses were performed using custom-written Spike2 scripts. Frequency responses were evaluated and fitted using the software Kernel, written by Dr Andrew French (Dalhousie University, Halifax, Canada). Statistical hypothesis tests were done using Matlab (The Mathworks, Natick, MA, USA).

To compare the latency–temperature relationship between walking and heated animals, we fitted linear mixed-effects models (LMM), accounting for differences in average latency and temperature response between individuals, using the ‘lme4’ package [22] in R v. 4.0.3. Prospective models were compared via likelihood-ratio tests, selecting models with lower Akaike information criterion [23]. *Post hoc* tests and estimated marginal means were calculated for the final model with the ‘emmeans’ package [24], using the Kenward–Roger method to estimate degrees of freedom.

### (f) Pulses

Responses to light pulses were evaluated by extracting the times of onset of the light pulses and the times when the ERG crossed a threshold of 50% of its maximum amplitude. The difference between the onset of each light pulse and the crossing of the threshold gave the latency. Latencies were averaged over 5 s (i.e. from five pulses) either from a phase prior to the onset of walking, during walking or after a recovery period of at least 5 min.

### (g) Cross-correlation

To measure the latency of the ERG during stimulation with Gaussian noise, we calculated the cross-correlation between the signal of the photodiode and the ERG. Cross-correlation measures the similarity of two waveforms in the time domain and can assume values between −1 and 1. A value of 1 indicates identical waveforms (except for amplitude), 0 indicates no correlation between the waveforms and −1 indicates identical waveforms (except for amplitude) with inverted signs. Cross-correlations were plotted as the above-described similarity value against the time that one signal needs to be shifted with respect to the other to obtain this similarity value. Therefore, the time-value at the minimum of the cross-correlation indicates the latency between the two signals.

During experiments, cross-correlations were continuously calculated to monitor the ERG latency. For evaluation, we chose segments of 5 s immediately preceding walking (or application of an external heat stimulus), immediately after walking (or heat stimulus) and upon recovery after at least 5 min. Walking activity often caused artefacts in the ERG recording, which made it necessary to choose a post-walking segment for evaluation, rather than the last 5 s of walking activity. Because of the slow time course of recovery, this had only minor influence on the results.

### (h) Coherence

Linear coherence was used to measure the similarity in frequency content between the white noise light stimulus measured by the photodiode and the ERG. At any given frequency, coherence is 1 if the relationship between input and output is purely linear, i.e. if input and output waveforms have a constant amplitude ratio and phase over the evaluated time period and the system is free of noise. Values smaller than 1 indicate either nonlinearity or added noise. The linear coherence function *γ*^2^(f) ranges between 0 and 1. It was calculated as follows:

$$\gamma^{2}(f)=\frac{\left\langle S_{xy}{\left(f\right)}^{2}\right\rangle }{\left\langle S_{xx}(f)\right\rangle \left\langle S_{yy}(f)\right\rangle }$$

where *S_xx_*(*f*) is the input spectrum (of the Gaussian white noise stimulus measured by the photodiode), *S_yy_*(*f*) is the output spectrum (of the ERG), *S_xy_*(*f*) is the cross-spectrum and ⟨⟩ indicates averaging over ensembles [25]. Spectra were obtained by resampling the data at 1 ms sampling intervals and calculating the fast Fourier transformation [26] using 512 point segments.

### (i) Gain and fitting

Frequency responses were obtained by direct spectral estimation from the cross-spectrum and the input spectrum [25] as follows:

$$G(f)=\frac{\left\langle S_{xy}(f)\right\rangle }{\left\langle S_{xx}(f)\right\rangle }$$

where *S_xx_*(*f*) is the input spectrum (of the Gaussian white noise stimulus as measured by the photodiode), *S_xy_*(*f*) is the cross-spectrum and ⟨⟩ indicates averaging over ensembles. The gain was fitted with a second-order low-pass filter, which was weighted by the coherence. The filter had the form:

$$H(f)=\frac{\beta }{1+j2\omega \tau \zeta \;-\;{\left(\omega \tau \right)}^{2}}$$

where *β* is amplitude, *τ* is a time constant, *ζ* is a damping factor, *ω* is radial frequency (2*π*f) and *j* is √(−1).

## 3. Results

### (a) Walking accelerates the electroretinogram response

We first measured the ERG in response to individual light pulses produced by a green (530 nm) LED (figure 1*b*; 50 ms duration at 1 Hz, pulse intensity: 2 × 10^15^ photons cm^−2^ s^−1^) prior to walking (sitting, light blue), during walking (violet) and after walking (sitting, dark blue) (figure 1*b*). We found that the time course of the ERG was shifted to the left during walking in comparison with the ERGs measured while the animal was sitting prior to and after walking. To quantify this shift, we measured the time between the onset of the stimulus and the time at which the ERG response crossed the half-maximum amplitude (figure 1*b*). Across all recordings (*n* = 16), the median latency at half-maximum was significantly shorter when the animals were walking (8.7 ms) than when they were sitting (before walking: 10.3 ms, after walking: 10.1 ms; figure 1*c*; before walking versus walking: *p* = 0.0003, after walking versus walking; *p* = 0.0003, before walking versus after walking: *p* = 0.1359, Wilcoxon test with Bonferroni correction (*α* = 0.0167)). This suggests a locomotor-dependent increase in visual response speed in walking bumblebees.

### (b) Increased visual response speed during walking coincides with increase in eye temperature

**Figure 2.**
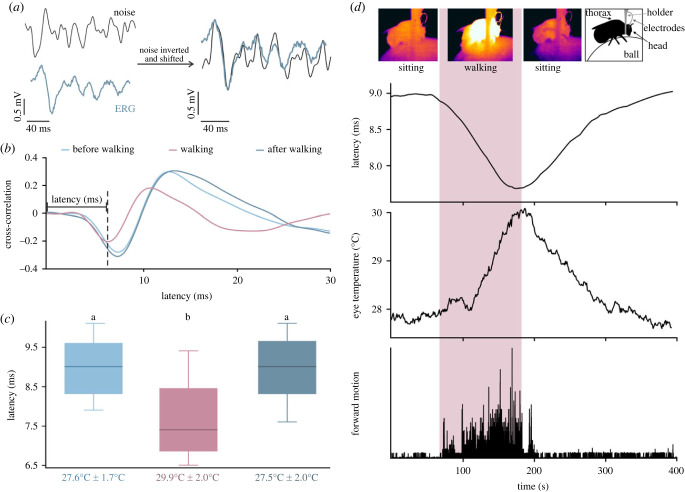
Effects of locomotion on the ERG of *B. terrestris* during stimulation with Gaussian white noise. (*a*) Section of the Gaussian noise stimulus (black; for whole trace, see electronic supplementary material, figure S1) and the corresponding ERG response (blue). To illustrate the similarity between noise stimulus and ERG response, the noise track was inverted and shifted (right). (*b*) Cross-correlation between the signal of the photodiode and the ERG to measure the latency of the ERG during stimulation with Gaussian noise. Cross-correlation measures the similarity of two waveforms in the time domain and can assume values between −1 (identical waveforms with inverted signs) and 1 (identical waveforms). The *x*-value at the minimum of the cross-correlation indicates the latency between the two curves. The minimum during walking (violet) occurs earlier than when the animal is sitting (blue). (*c*) Boxplots show the responses of 12 animals to the noise stimulus, before walking (light blue; 27.6°C ± 1.7°C), during walking (violet; 29.9°C ± 2.0°C) and after walking (dark blue; 27.5°C ± 2.0°C). The latency (ms) was significantly shorter during walking than during sitting (*p* < 0.0005, Wilcoxon test with Bonferroni correction), but no differences were seen when comparing before and after walking (*p* = 0.748, Wilcoxon test with Bonferroni correction). Boxplots show median, IQR, whiskers with 1.5 x IQR and outliers greater than 1.5 x IQR. (*d*) Time course of latency (upper trace), temperature (middle trace) and forward motion (lower trace). During locomotion (highlighted in violet), the latency decreased and the temperature increased (symbolized with thermographic pictures at the top; schematic drawing shows the position and orientation of the animal). After walking, the values return to their initial level.

To be able to assess the frequency range of the ERG response that was particularly affected by walking, we repeated our experiments, this time using a Gaussian white noise stimulus (figure 2*a*, Methods and electronic supplementary material for details). This allowed us to continuously monitor the cross-correlation between the stimulus signal and the ERG as well as to compute the frequency response function. The cross-correlation computes the similarity of two curves, and its minimum (or maximum) indicates the relative shift of the two curves, i.e. the latency between them. Figure 2*b* displays example traces of cross-correlation curves showing that the latency was shorter during walking (violet, 6.5 ms) than during sitting both before walking and after walking (light and dark blue, both 8.3 ms). This observation was confirmed in all tested animals, and the difference in latency was significant (figure 2*c*; *n* = 12; median values: before walking, 9.0 ms, walking, 7.4 ms, after walking, 9.0 ms; before walking versus walking: *p* = 0.00048, after walking versus walking: *p* = 0.00048, before walking versus after walking: *p* = 0.748, Wilcoxon test with Bonferroni correction (*α* = 0.0167)).

To test if the changes in visual processing speed can be explained by changes in temperature, we used a thermographic camera to track the temperature of the bumblebee's compound eyes while sitting and during walking. We found that during walking, the temperature of the animals' eyes increased in conjunction with the heating of their thorax, and then decreased again when the animals were sitting (figure 2*d*; electronic supplementary material, figure S2, Video). Comparison of the changes in latency and temperature revealed that both effects occurred during walking and slowly decreased as soon as the animal stopped walking. These temperature latency correlations applied to all animals tested (figure 2*c*).

**Figure 3.**
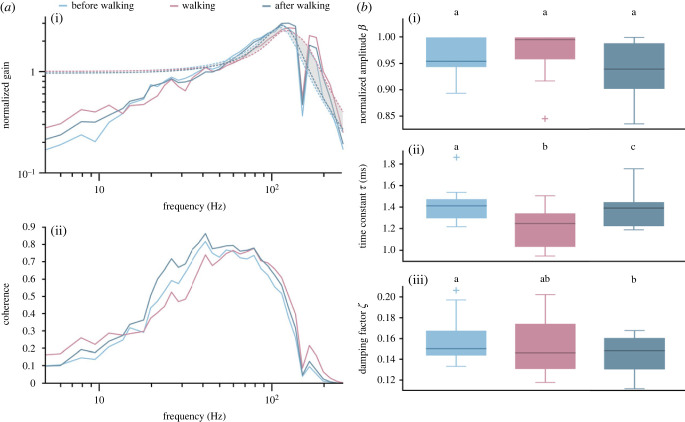
Gain and linear coherence between the stimulus and the ERG for experiments with walking bumblebees. (*a*) To assess which frequency range of the ERG response was specifically affected, we calculated gain (i) and linear coherence (ii) and averaged over 11 animals (for individual responses see electronic supplementary material, figures S3 and S4). The gain was fitted with a second-order low-pass filter (dashed lines). During walking, a shift to higher frequencies was observed (grey shaded area indicates right-shift of fit). Dips at 150 Hz result from a noise artefact that could not be eliminated. (*b*) Parameters of second-order low-pass fit to the gain. The amplitude *β* did not differ between any of the conditions ((i); *p* > 0.05, Wilcoxon test with Bonferroni correction). The time constant *τ* became significantly smaller during walking ((ii); *p* = 0.001, Wilcoxon test with Bonferroni correction). There was a small, but significant difference in *τ* between the two sitting conditions ((ii); *p* = 0.014, Wilcoxon test with Bonferroni correction). Walking had no effect on the damping factor *ζ* ((iii); *p* > 0.1, Wilcoxon test with Bonferroni correction), but there was a small, but significant difference between the two sitting conditions ((iii); *p* = 0.004, Wilcoxon test with Bonferroni correction). Boxplots show median, IQR, whiskers with 1.5 x IQR and outliers greater than 1.5 x IQR.

To evaluate the frequency range of the ERG response that was specifically affected, we calculated the gain and the linear coherence (figure 3*a*, *n* = 11) between the stimulus and the ERG. Gain is a measure of how strong an output signal (here: ERG) is compared with the input signal (here: Gaussian white noise light stimulus), at each frequency in the signal. Linear coherence is a value between 0 and 1 that measures the similarity in frequency content of two signals. It is 1, if the relationship between the input and the output (ERG) is completely linear and no noise is added by the system (i.e. the visual system and the measuring devices). We found that both gain and coherence were on average shifted to higher frequencies during walking (figure 3*a*). In some recordings, such a shift was not observed and this was more likely in animals that heated up less during walking (electronic supplementary material, figure S7, left). To quantify the frequency-dependent changes that occurred during walking, we fitted second-order low-pass filter functions to the gain (see Methods for details). The time constant *τ* of this filter determines at which frequency the filter function rolls off such that smaller values of *τ* indicate a shift to higher roll-off frequencies. Comparing the median time constants during sitting and walking, we found that *τ* became significantly smaller during walking (figure 3*b*ii; *n* = 11; median values: before walking, 1.41 ms, walking, 1.25 ms, after walking, 1.39 ms; before walking versus walking: *p* = 0.001, after walking versus walking: *p* = 0.001, before walking versus after walking: *p* = 0.014, Wilcoxon test with Bonferroni correction (*α* = 0.0167)). We also found a small, but significant difference in *τ* between the two sitting conditions, which probably indicates that the system had not completely recovered from the walking condition. Walking had no effect on the damping factor *ζ*, but as for *τ*, there was a small, but significant difference in the values of *ζ* between the two sitting conditions (figure 3*b*iii; *n* = 11; median values: before walking, 0.150, walking, 0.146, after walking, 0.148; before walking versus walking: *p* = 0.563, after walking versus walking: *p* = 0.123, before walking versus after walking: *p* = 0.004, Wilcoxon test with Bonferroni correction (*α* = 0.0167)). The amplitude *β* of the fit did not differ between any of the conditions (figure 3*b*i; *n* = 11; median of normalized values: before walking, 0.955, walking, 0.996, after walking, 0.939; before walking versus walking: *p* = 0.700, after walking versus walking: *p* = 0.064, before walking versus after walking: *p* = 0.320, Wilcoxon test with Bonferroni correction (*α* = 0.0167)).

### (c) Experimental increase of eye temperature replicates effects of walking

**Figure 4.**
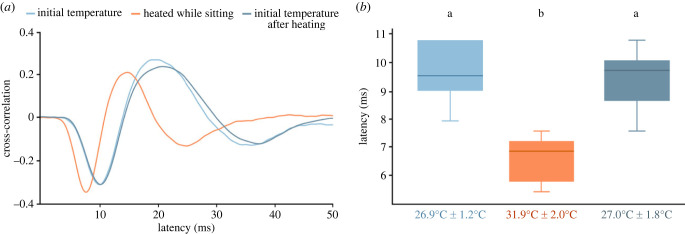
Effects of temperature on ERGs of photoreceptor cells in bumblebees stimulated with noise stimulus. (*a*) Cross-correlation between the signal of the photodiode and the ERG to measure the latency of the ERG during stimulation with Gaussian noise. The minimum of the cross-correlation occurred earlier, when the bumblebee was heated up while it was sitting (orange) compared with when the animal was just sitting (shades of blue). (*b*) Boxplots show the responses of 22 animals to the noise stimulus, before heating (light blue; median temperature: 26.9°C ± 1.2°C), while heated (orange; 31.9°C ± 2.0°C) and after return to initial temperature (dark blue; 27.0°C ± 1.8°C). The latency was significantly shorter when heated than at the initial temperature (*p* = 3 × 10^−5^, Wilcoxon test with Bonferroni correction), but no differences were seen when comparing the initial temperatures before and after heating experiments (*p* = 0.0534, Wilcoxon test with Bonferroni correction). Boxplots show median, IQR, whiskers with 1.5 x IQR and outliers greater than 1.5 x IQR.

To test whether the changes in temperature of the compound eyes during walking were sufficient to explain the observed changes of the visual processing speed, ERG recordings using Gaussian white noise stimulation were carried out as before, but this time the animals were not walking during the experiment. Instead, their head temperature was experimentally raised up to 37°C using an infrared heating lamp. Again, the cross-correlation between the Gaussian white noise stimulus, and the ERG was calculated to determine the latency between the two signals. The example cross-correlations in figure 4*a* illustrate that the latency in the heated condition was shorter (orange; 7.2 ms) compared with the initial temperature before heating and when returning to that temperature after heating (light and dark blue; both 10.8 ms). The median latency of all animals was significantly shorter (figure 4*b*; *n* = 22; before heating versus heating: *p* = 3.78 × 10^−5^, after heating versus heating: *p* = 3.63 × 10^−5^, before heating versus after heating: *p* = 0.0534, Wilcoxon test with Bonferroni correction (*α* = 0.0167)) when the animals were heated up (31.9°C ± 2.0°C) than when they had their initial temperatures (before: 26.9°C ± 1.2°C; after: 27.0°C ± 1.8°C).

**Figure 5.**
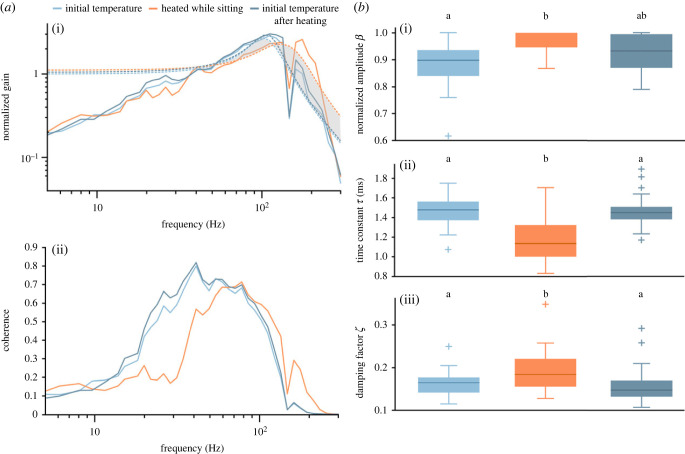
Gain and linear coherence between the stimulus and the ERG for experiments in which the bumblebees were heated by an infrared lamp*.* (*a*) Averaged gain and coherence showed a clear shift to higher frequencies in heated animals (*n* = 22; for more details see electronic supplementary material, figures S5 and S6). Dashed lines are fits of second-order low-pass filter functions. In the heated condition, a shift to higher frequencies was observed (grey shaded area indicates right shift of fit). Dips at 150 Hz result from a noise artefact that could not be eliminated. (*b*) Parameters of second-order low-pass filter fits. The amplitude *β* of the fit function increased significantly during heating compared with the condition before heating (*p* = 0.0012, Wilcoxon test with Bonferroni correction), whereas there were no significant differences in the amplitude between the sitting conditions and between the heating condition and the after heating condition (*p* > 0.02, Wilcoxon test with Bonferroni correction). The time constant *τ* was significantly smaller during heating compared with both sitting conditions (*p* < 0.0001, Wilcoxon test with Bonferroni correction), but showed no significant difference between the sitting conditions (*p* = 0.592, Wilcoxon test with Bonferroni correction). The damping factor *ζ* was significantly increased during heating compared with both sitting conditions (*p* ≤ 0.001, Wilcoxon test with Bonferroni correction), while it did not differ between the sitting conditions (*p* = 0.287, Wilcoxon test with Bonferroni correction). Boxplots show median, IQR, whiskers with 1.5 x IQR and outliers greater than 1.5 x IQR.

Furthermore, gain and coherence showed a clear shift to higher frequencies in heated animals (figure 5*a*). The time constant *τ* of the second-order low-pass filter that was fitted to the data was significantly smaller during heating compared with both sitting conditions (figure 5*b*ii; *n* = 22; median values: before heating, 1.48 ms, heating, 1.14 ms, after heating, 1.45 ms; before heating versus heating: *p* < 0.0001, after heating versus heating: *p* < 0.0001, before heating versus after heating: *p* = 0.592, Wilcoxon test with Bonferroni correction (*α* = 0.0167)). The damping factor *ζ* was significantly increased during heating compared with both sitting conditions (initial temperature before and after heating), while it did not differ between the initial temperature conditions (figure 5*b*iii; *n* = 22; median values: before heating, 0.165, heating, 0.184, after heating, 0.148; before heating versus heating: *p* = 0.0003, after heating versus heating: *p* = 0.0010, before heating versus after heating: *p* = 0.287, Wilcoxon test with Bonferroni correction (*α* = 0.0167)). Heating of the animals also increased the amplitude *β* of the fit function significantly compared with the before heating condition (sitting with initial temperature). The amplitude between the two sitting conditions (before and after heating) and the heating compared with sitting after heating showed no significant difference (figure 5*b*i; *n* = 22; median of normalized values: before heating, 0.897, heating, 1.000, after heating, 0.932; before heating versus heating: *p* = 0.0012, after heating versus heating: *p* = 0.0309, before heating versus after heating: *p* = 0.0261, Wilcoxon test with Bonferroni correction (*α* = 0.0167)).

To statistically assess if temperature changes are sufficient to explain the increase in visual processing speed in walking bumblebees, we evaluated the latency between stimulus and response at five different eye temperatures of walking and externally heated bumblebees.

As predicted, there was a strong negative relationship between temperature and latency (latency = 22.21–0.48 ms °C^−1^; AIC: 492.34 versus 129.89, Δdeviance = 368.46, d.f. = 3, *p* < 0.0001). This relationship did not differ significantly between recordings from walking and heated animals (AIC: 129.89 versus 130.40, Δdeviance = 1.4823, d.f. = 1, *p* = 0.2234), although an effect of treatment (AIC: 133.46 versus 129.89, Δdeviance = 5.5753, d.f. = 1, *p* = 0.01822) indicates that recordings in which animals were heated had a slightly, but significantly longer latency on average (mean ± s.e: heated walking = 177 ± 76.8 µs s.e. slower; LMM, *t* = 2.303, d.f. = 114, *p* = 0.0231). The origins of this small offset between the two groups are currently unknown. However, in the observed range of latencies (5.4–10.1 ms), it only accounts for 1.8%–3.3% of the total offset. Our data therefore show that temperature is sufficient to explain the changes in latency observed in walking bumblebees.

It should be noted that, while the linear regression describes the relationship between temperature and processing speed well in our dataset, this is probably only true for a limited temperature range while the effect probably saturates at higher temperatures.

### (d) Walking accelerates visual responses as much as a 14-fold increase in light intensity

Another factor that can increase the processing speed of photoreceptors is adaptation to higher light intensities [27]. We therefore asked how the walking-induced increase in processing speed (from 9.0 to 7.4 ms) compares with the increase in processing speed that is induced by adaptation to higher light intensities.

**Figure 6.**
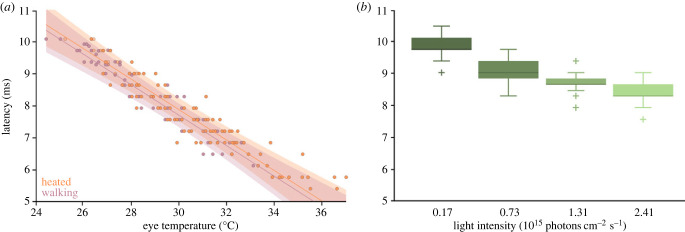
Comparison of temperature and intensity effects. (*a*) Latency for walking (violet; 60 data points from 12 animals) and heated (orange; 110 data points from 22 animals) animals plotted against the eye temperature. With increasing temperature, the latency of the ERG decreases. Linear model predictions for the final model (fixed effects of temperature and treatment, individual effects on latency and temperature response) are shown as lines, with bootstrapped 95% confidence intervals represented by shaded areas (for more details, see electronic supplementary material, figures S8 and S9). (*b*) The latency of ERG responses from 17 animals was measured after adaptation to four different intensities (1.74 × 10^14^, 7.27 × 10^14^, 1.31 × 10^15^ and 2.41 × 10^15^ photons cm^−2^ s^−1^). As the light intensity increases, the latency decreases. Boxplots show median, IQR, whiskers with 1.5 x IQR and outliers greater than 1.5 x IQR.

To test this, we presented the Gaussian white noise stimulus to sitting bumblebees at four different light intensities (1.74 × 10^14^, 7.27 × 10^14^, 1.31 × 10^15^ and 2.41 × 10^15^ photons cm^−2^ s^−1^). As expected, adaptation to higher light intensities led to a shorter latency between stimulus and response (figure 6*b*). The difference in latency between the highest and the lowest light intensity tested was 1.4 ms. In comparison, locomotion decreased the latency between stimulus and response by 1.6 ms (figure 6). In other words, walking increased the processing speed more than a 14-fold increase in light intensity did (figure 6). This strong effect is especially impressive, when considering that the median observed temperature change during walking was only 2.3°C. It is known that the head temperature of bumblebees in flight can rise up to 35°C [28]. From our data, we conclude that the latency between stimulus and ERG in a flying bumblebee would be 5.7 ms, i.e. 3.3 ms faster than during sitting (figure 6*a*).

## 4. Discussion

We show here that the ERG response of the bumblebee's compound eye is accelerated during walking and that this increase in visual processing speed is caused by a rise of the compound eye's temperature. The observed temperature changes of the eyes are tightly linked to temperature changes in the thorax (electronic supplementary material, figure S2), where the flight muscles are used to generate heat. While the idea that active heat production could be used to increase the temporal resolution of the compound eyes has been suggested more than 20 years ago [18], our study provides the first direct experimental evidence showing that the heat that an insect produces in its thorax during walking is indeed sufficient to effectively speed up the visual system.

Studies in flies have shown that the behavioural state increases the gain of motion-sensitive visual interneurons [9,10]. These changes are mediated through the release of octopamine by modulatory interneurons [12,13]. Recently, it was shown that octopamine signalling is also necessary for shivering thermogenesis in the flight muscles of honeybees [29], and this is likely to also apply to bumblebees. Like the gain increase in visual interneurons, the increase in photoreceptor processing speed is therefore likely to also involve the action of octopamine. While octopamine acts through very different pathways in these two cases, the resulting action is always an adjustment of the visual system to the needs of the current locomotor state.

Neurophysiological processes depend on temperature because electrochemical neuronal potentials are established by asymmetric distribution of ions across the cell membrane. It is therefore not surprising that sensory processes are temperature dependent. However, the way in which temperature influences sensory processes is modality dependent. For example, in mechanoreceptors of cockroaches and spiders, higher temperatures lead to an increase in response gain that is independent of stimulus frequency [30,31]. This means that a temperature change, which doubles the gain at a low stimulus frequency, will also double the gain at high frequencies, but the range of frequencies that the receptor responds to is not changed. Photoreceptors, on the other hand, usually become more sensitive to higher frequencies with increasing temperature [15,18,32]. Higher temperatures of a receptor can be either caused by a rise in ambient temperature or through production of heat by the insect. As locomotion speed of insects is usually positively correlated with ambient temperature, both cases are usually linked to a higher locomotion speed. While locomotion inevitably increases the range of temporal frequencies that the eyes have to process, it is unlikely to have a strong effect on the frequency range which mechanoreceptors might be exposed to. Physiological changes that receptor systems undergo during temperature changes, seem therefore be matched to the behavioural needs of the animals. It is therefore conceivable that walking (and flying) insects actively increase the temperature of their eyes in order to adjust the frequency range of their receptors to the sensory needs. Visually guided flight requires an even faster response of the compound eyes than walking. Reber *et al*. [28] found head temperatures of up to 35°C in flying bumblebees, suggesting even faster visual responses than the ones we measured in walking animals. While the activity of flight muscles always leads to heat production in the thorax, it should be mentioned that the blood flow to the head, and therefore its temperature, can be actively regulated [19].

Active increase of eye (and brain) temperature has been previously shown in large pelagic fish, like marlins, sailfish and sawfish. These species can swim in cold water and have developed a special tissue to heat up their eyes about 10°C above the ambient temperature [20,33]. In the swordfish retina, such a rise in temperature effectively increases the processing speed of visual stimuli in the retina by a factor of 5.2 when the eye is heated up by 10°C [21]. It is assumed that active heating of the eyes gives these predatory fish an advantage over their prey, when hunting in cooler waters. Similar to the mechanism in sawfish, heat production of bumblebees during walking could be an active way of tuning their eyes to the sensory requirements of the current behaviour.

Faster processing of sensory information at the receptor level will only be useful to the animal if the subsequent neuronal circuitry is also adapted to handle the faster incoming information. While our observations in bumblebees pertain only to the primary sensory processing of visual information, heating of the head will necessarily increase the temperature of the brain as well and thus effect any aspect of neuronal processing. It is well documented through electrophysiological recordings from visual interneurons in insects, that a rise in temperature leads to a decrease in response latency [34,35] and an increase in firing rate [36]. Of course, such effects are not limited to the visual system, as temperature affects basic neuronal properties like the input resistance, the membrane time constant and hence the conduction velocity, as well as synaptic transmission [37,38]. The overall effect of temperature on complex neuronal circuits is hardly predictable. For example, central pattern generators (CPGs) in the same animal can respond very differently to changes in temperature. While the locust flight CPG is almost completely temperature compensated, the locust ventilation CPG increases its frequency 2.3-fold when temperature rises by 10°C [39]. It would therefore be desirable for future neuroethological electrophysiology experiments to take the natural temperature range that is linked to the behaviour under investigation into account, whenever possible.

When we compared the effect of temperature and adaptation on the increase in response speed, we found that walking had a similar effect as a 14-fold increase in light intensity. While our experiments were done with rather high light intensities on light-adapted animals, bumblebees are known to be able to also fly under dim light conditions [28]. However, light levels seem to be a limiting factor for the flight velocity of the bumblebees. The darker the ambient light levels, the slower the velocity of the animals and the more tortuous their flight paths [28]. The ability to fly at all at low light levels might be a result of the ability to heat, and hence speed up the visual system, to compensate for a reduction in processing speed that is necessitated by longer integration times in dim light.

Our findings that heat produced during walking accelerates the visual system of bumblebees are very likely to be also of profound significance to many other insect species that are capable of temperature regulation [19]. It will therefore be interesting to see how increased head temperatures during walking or flying influence the visual system and the neuronal responses of other insect species like large moths, beetles or dragonflies.

## Data Availability

Raw data and analyses scripts are available from the Dryad Digital Repository: https://doi.org/10.5061/dryad.zs7h44jf6 [40]. Additional figures and a video can be found in the electronic supplementary material [41].
